# 

**Published:** 1883-06-20

**Authors:** 


### EDITORIALS AND MISCELLANEOUS.

### *EDITORIAL NOTICES.*

ANGLO-SWISS MILK FOOD.—See the new advertisement of this excellent food preparation, commencing with this number of our Journal.

SOUTHERN MEDICAL COLLEGE, ATLANTA.—See the advertisement of this new and rapidly rising school, commencing with this number of our Journal.

BEEF PEPTONOIDS.—We received, through the courtesy of Messrs. Reed & Carnrick, a sample of Beef Peptonoids for trial. We find it a very convenient and excellent preparation. See the advertisement of this article in this Journal.

COLLEGE ADVERTISEMENT.—See the advertisement of the University of Pennsylvania Medical Department, commencing with this issue of our Journal, an old and deservedly popular Institution.

AMERICAN MEDICAL ASSOCIATION.—A letter from an esteemed correspondent giving a sketch of the late meeting of the American Medical Association, at Cleveland, Ohio, was received too late for insertion in this issue of our Journal. It will appear in our next.

GOOD LOCATION FOR A PHYSICIAN.—A practitioner residing in Southeastern Georgia, offers to sell his home, resigning a fine field of practice to the purchaser. His location is an excellent one, and his patrons good pay. See his card under the Special Notice head.

AHL'S SPLINTS.—A full set of these Splints, sold at $30 by the proprietors, may be bought at $25 of A. L. Hernstein, Surgical Instrument dealer, Atlanta, Georgia. With these Splints on hand the physician is ready at a moment's notice to dress any sort of fracture. The Splints are new, and have not been used. He has also an Obstetric Case of Instruments (second hand but not injured) which can be bought at low figures, ($10).

WYETH'S TABLETS.—We have received, through the courtesy of John Wyeth & Brothers, Philadelphia, beautiful samples of their superb preparations. Among them a specimen of their soda tablets, the neatest we have seen. We no longer fear that troublesome affliction of sedentary life, the heartburn, as we have only to swallow one of Wyeth's soda tablets and it is instantly cured.

OFF FOR THE SPRINGS.—Our Senior editor, Prof. Thos. S. Powell, has decided to take a temporary refuge from the arduous duties of the regular practice by visiting the Blue Ridge Springs of Virginia, and spending the months of July and August, and yet, as he has consented to act as resident physician of this great health resort, he may not find the full measure of rest that he anticipates. Almost any change, however, must prove pleasant to the care-worn physician who escapes, even for a brief period, from the responsible and incessant labors of an extensive practice. W.

SOUTHERN WORLD.—This is an Agricultural Paper, published in Atlanta, and extensively circulated in the Southern States. For the compliment voluntarily paid our Journal and its managing editor in a late issue of *The World* we return our sincere thanks. The *Southern World* is an ably conducted and deservedly popular paper, neatly gotten out and beautifully illustrated. Dr. B. M. Woolley, proprietor; W. P. Woolley, manager, and W. G, Whidby, editor—all intelligent, enterprising and clever gentlemen.

### *RECEIPTED.*

1882.—Drs. T W Spruell, James Tatum, Elias Goddard, T J Small, E Y Larkin, Samuel T Jones, Robert Shaffin, Tho R Littleton.

1883.—Drs. B M Woolley, W Delbridge, J A Agnew, W P Anderson, J L Hamilton, M D Miles, C. M Bold, F P H Akers, T Swanton.

